# Can neuro-oncology teaching contribute to educate medical doctors better? A reflection on the value of neuro-oncology for student teaching

**DOI:** 10.1186/s41016-022-00293-1

**Published:** 2022-09-02

**Authors:** Matthias A. Mäurer, Irina Mäurer, Marcel A. Kamp

**Affiliations:** 1 Department of Radiation Oncology, University Medical Center Jena, Hospital, Friedrich-Schiller-University Jena, Bachstraße 18, 07743 Jena, Germany; 2 Clinician Scientist Program “OrganAge”, Jena University Hospital, 07747 Jena, Germany; 3 Department of Neurology, Jena University Hospital, Jena, Germany; 4 Else Kröner Research School for Physicians “AntiAge”, Jena University Hospital, 07747 Jena, Germany; 5 Department of Neurosurgery, Centre of Neuro-Oncology, Jena University Hospital, Friedrich-Schiller-University Jena, Am Klinikum 1, 07747 Jena, Germany; 6 Centre of Neuro-Oncology, Department of Neurosurgery, Medical Faculty, Heinrich-Heine-University Düsseldorf, Moorenstraße 5, 40225 Düsseldorf, Germany

**Keywords:** Neuro-oncology, Education, Framework, Medical studies, Digital learning

## Abstract

Neuro-oncology, with its various conservative, surgical, and interventional disciplines, is ideally suited to teach basic knowledge, skills, and attitudes important to medical practice in general. However, training is less about teaching specific treatment protocols and more about fostering skills for interdisciplinary collaboration, development of treatment recommendations, communication skills, and an ethical stance. To adequately teach this content, new and innovative formats are needed to test and learn high levels of student interaction, communication, and collaboration.

New teaching concepts such as inverted teaching formats as well as the use of modern media technology can be helpful to improve networking between disciplines and to improve the quality of medical education.

Dear Editor,

Neuro-oncology is increasingly developing into an independent and complex branch within neurosurgery, neurology, and radiation oncology. Neurological, neurosurgical, oncological, and radio-oncological education are each an integral part of medical education curriculum. In contrast, neuro-oncological teaching is often not a topic in regular medical frameworks and even specialties regularly treating neuro-oncological patients often do not sufficiently address neuro-oncological themes [1]. This raises the question of whether there is a place for multidisciplinary neuro-oncology teaching in the medical curriculum and what the benefits would be.

We strongly believe that student training concepts could benefit from teaching neuro-oncology because, as a cross-sectional field, it offers the opportunity to teach competencies that are essential for general medical practice. Therefore, we would like to highlight some of these aspects:

First, complex (neuro-)oncology therapeutic plans can no longer be drawn up by individual doctors or specialist disciplines. Good (oncological) medicine is undoubtedly a team task that requires the involvement of different disciplines. Therefore, students should learn how to take an active and considerate role in an interdisciplinary medical team. Since neuro-oncology is genuinely a sub-area of several disciplines, and thus, to a large extent a cross-sectional subject, it is particularly suited to introduce students to interdisciplinary collaboration.

Second, the subject area of neuro-oncology is ideally suited to teach the use of scientific and evidence-based sources and the creation of individual therapy plans. As an innovative, interdisciplinary field, neuro-oncology is characterized by continuous advancements in the precision of surgical or radiotherapeutic procedures or the increasing use of targeted therapies. Using the example of neuro-oncological disease patterns, students can learn how to create evidence-based therapy plans and use other sources (best medical practice, expert opinion) where there is no evidence.

Third, modern medicine and in particular neuro-oncology highly involves ethical issues. Basic principles of medicine ethics such as respect of autonomy, beneficence, non-maleficence, and justice are taught in medical schools. Neuro-oncology involves further practical medical-ethical questions daily [2]. These include the tension between prolonging life with specific anti-tumor therapies and potentially compromising quality of life. We are convinced that discussion of these issues is highly relevant to become a capable physician and to acquire a responsible attitude in addition to the acquisition of knowledge and skills.

Fourth, interdisciplinary cooperation requires a clear and precise communication about (critically ill) patients. Advise of patients and their relatives on neuro-oncological diseases, their symptom load, prognosis, operative, and non-operative therapies are essential medical tools. This includes the professional delivery of bad news and an activation of patient’s individual, familial, and social resources to enable them to cope with the situation of a life-threatening illness. Therefore, the knowledge and application of standard and specific communication strategies as required in neuro-oncology should be integral part of all medical frameworks.

Fifth, neuro-oncology reflects the enormous technological progress in medicine. This includes personalized therapy approaches, the use of big data analyses or innovative imaging systems (e.g., radiomics), artificial intelligence, or the use of mixed reality during neuro-oncological surgeries or for radiation planning. Modern oncology medicine combines histo-pathological, molecular and epigenetic, imaging, and clinical and social information with modern technologies. These forward-looking approaches are relevant to the entire medicine, but their interdisciplinary use manifests itself particularly in the neuro-oncology setting. This cross-sectional area is therefore particularly well suited to illustrate what the medicine of the future will look like and how modern techniques and integration of data might be used to improve patient’s outcomes.

Sixth, digital modalities are routinely used in many neuro-oncology teams. The safe use of digital platforms is a core competence for all medical school graduates, as these tools will become increasingly important for optimal collaboration between (inter-)national research groups and clinical experts or in patient care in the future.

Neuro-oncology, with its various conservative, surgical, and interventional disciplines, is ideally suited to teach basic knowledge, skills, and attitudes important to medical practice in general. However, training is less about teaching specific treatment protocols and more about fostering skills for interdisciplinary collaboration, development of treatment recommendations, communication skills, and an ethical stance. To adequately teach this content, new and innovative formats are needed to test and learn high levels of student interaction, communication, and collaboration. New teaching concepts, such as inverted teaching formats or the development of Entrustable Professional Activities (EPAs), can be helpful, as can the use of modern media technology. They can lead to better networking between disciplines and to an improvement in the quality of medical education [3].

Multidisciplinary and -professional evaluation, guideline-based but individualized treatment plans, imaging data and clinical big data analysis are the future development direction of neuro-oncology and an important way for students to master the frontier of neuro-oncology. The authors advocate for increased integration of neuro-oncology into medical curricula, as qualified and adequate neuro-oncology education can help to better train physicians overall.

## Data Availability

The manuscript has no associated data.
